# Identifying patients with undiagnosed small intestinal neuroendocrine tumours in primary care using statistical and machine learning: model development and validation study

**DOI:** 10.1038/s41416-024-02736-1

**Published:** 2024-06-03

**Authors:** Ash Kieran Clift, Hadley Mahon, Ghazanfar Khan, Freya Boardman-Pretty, Amanda Worker, Elena Marchini, Orlando Buendia, Peter Fish, Mohid S. Khan

**Affiliations:** 1 Mendelian, The Trampery Old Street, London, UK; 2 South Wales Neuroendocrine Cancer Service, University Hospital of Wales, Cardiff and Vale University Health Board, Heath Park, Cardiff, UK; 3 Cardiff University, School of Medicine, University Hospital of Wales, Cardiff, UK

**Keywords:** Epidemiology, Risk factors

## Abstract

**Background:**

Neuroendocrine tumours (NETs) are increasing in incidence, often diagnosed at advanced stages, and individuals may experience years of diagnostic delay, particularly when arising from the small intestine (SI). Clinical prediction models could present novel opportunities for case finding in primary care.

**Methods:**

An open cohort of adults (18+ years) contributing data to the Optimum Patient Care Research Database between 1st Jan 2000 and 30th March 2023 was identified. This database collects de-identified data from general practices in the UK. Model development approaches comprised logistic regression, penalised regression, and XGBoost. Performance (discrimination and calibration) was assessed using internal-external cross-validation. Decision analysis curves compared clinical utility.

**Results:**

Of 11.7 million individuals, 382 had recorded SI NET diagnoses (0.003%). The XGBoost model had the highest AUC (0.869, 95% confidence interval [CI]: 0.841–0.898) but was mildly miscalibrated (slope 1.165, 95% CI: 1.088–1.243; calibration-in-the-large 0.010, 95% CI: −0.164 to 0.185). Clinical utility was similar across all models.

**Discussion:**

Multivariable prediction models may have clinical utility in identifying individuals with undiagnosed SI NETs using information in their primary care records. Further evaluation including external validation and health economics modelling may identify cost-effective strategies for case finding for this uncommon tumour.

## Introduction

Neuroendocrine neoplasms (NENs) are relatively uncommon, encompassing neuroendocrine tumours (NETs) and more aggressive neuroendocrine carcinomas (NECs). They present several clinical challenges: both their symptomatology and clinical behaviour can be highly heterogeneous, and their incidence is increasing [1, 2]. With an incidence of 8.6 per 100,000 per year, NETs can be difficult to diagnose, with NETs arising within the ‘midgut’ (i.e. small intestine) often diagnosed at advanced stages impacting survival [3–5]. In the absence of screening programmes, using clinical prediction tools embedded within electronic healthcare record software could support ‘case finding’, and identify enriched populations that could be suitable for specialist referral and testing.

Observational evidence reinforces clinical experience that patients with small intestinal NETs (SI-NETs) can experience diagnostic delay and misdiagnosis prior to the ultimate diagnosis of their condition [6]. In one retrospective survey-based study of 303 individuals with NET, the median time from first symptoms to eventual diagnosis was 36 months for SI-NET; 29% of these individuals received an initial diagnosis of irritable bowel syndrome [6]. Another survey-based study reported a mean duration of 60.1 months (range 0 to 300 months) from symptom initiation to SI-NET diagnosis, a mean of 10 appointments in primary care for their symptoms, and a mean time of being under investigation in primary care of 40 months [7].

The broad symptomatology of SI-NETs parallels the heterogeneity in tumour behaviour—florid manifestations such as the carcinoid syndrome (a harbinger of advanced disease with functional NETs) may co-exist with less archetypal manifestations within temporally dynamic symptom profiles of non-functional NETs [6, 7].

Whilst some tumours present ‘incidentally’, there is evidence that there may be a protracted time period during which vague, non-specific symptoms may be present and recorded in clinical records [6, 7]. This known, but incompletely characterised ‘symptomatic window’ may present opportunities for the development of novel case-finding techniques. Given the protracted duration of time that these symptoms may be present prior to eventual diagnosis [6, 7], there is the possibility that case finding for this rare tumour type could aid earlier detection. Other salient considerations include potential misattribution of symptoms to other, non-NET causes (e.g. bloating ascribed to irritable bowel syndrome), and ‘counterintuitive informative presence’ of some clinical events (e.g. the fact of a negative coeliac screen suggesting symptomatology due to an as-yet unknown cause). Such ‘negative findings’ may actually hold predictive utility in case-finding, as they reflect aspects of the diagnostic odyssey. Statistical or machine learning methods could be suitable for novel case-finding approaches. Whilst there is increasing interest in the potential for machine learning approaches to clinical prediction modelling, some have raised concerns about model transparency [8, 9], methodological limitations [8], and fairness of comparisons with classical techniques [10, 11], and there is no a priori way to understand which approach may be most appropriate for a given prediction task.

This study sought to develop and evaluate the performance and clinical utility of novel clinical prediction models, derived using statistical and machine learning techniques using large-scale, anonymised, primary care electronic healthcare record (EHR) data, that could identify patients at high risk of harbouring an as-yet undiagnosed SI-NET. The envisioned use cases (or points of use) of these tools is a clinical practitioner estimating the risk of a patient harbouring an as-yet undiagnosed SI-NET, using information currently available in their EHR, or a model running ‘passively’ in the EHR back-end.

## Methods

### Overall modelling strategy

This study evaluated four different model-building approaches—logistic regression, two penalised regression methods (LASSO logistic model, and ridge logistic model), and XGBoost. Within a diagnostic modelling framework, models were developed to provide a probabilistic estimate that a given individual had a SI-NET. Model evaluation was then performed using internal-external cross-validation [12].

### Minimum sample size calculation

The approach of Riley et al. for prediction models with a binary outcome was used to estimate the minimum sample size required [13]. Prior to the study commencing, a SQL query in the target database identified 709 recorded SI-NET cases. Using this number, and the size of the entire potential cohort at that time (*n* ~ 15,000,000), an outcome prevalence of 0.0047% was estimated. Assuming this, targeting an *R*-squared of 0.00015 (conservative 15% of the maximum permitted in this setting, 0.001), shrinkage of 0.9, and 50 predictor parameters, we required a dataset comprising 2,999,750 adults (141 cases, events per predictor parameter = 2.82). No clear guidance exists for estimating the minimum sample size for machine learning models.

### Study population and data sources

This study used the Optimum Patient Care Research Database (OPCRD) database (https://www.opcrd.optimumpatientcare.org), which had at the time, collected de-identified electronic routine primary care data from over 17 million patients registered at over 1000 general practices in the United Kingdom (UK). OPCRD collects data from practices using all UK clinical software systems. The data fields available in OPCRD include demographics, clinical encounters such as measurements and diagnoses (defined as the presence of recorded SNOMED and Read/CTV3 codes), prescriptions and referrals to secondary care.

An open cohort of adults registered with general practices contributing data to OPCRD between 1st Jan 2000 and 30th March 2023 was identified. Follow-up started from the latest of: cohort start date, date of registration with the practice plus 1 year (to exclude ‘temporary patients’), or the patient’s 18th birthday. Follow-up was until the earliest of NET diagnosis (for cases), date of leaving the practice/death, date of 90th birthday, or the cohort end date. Individuals that had a diagnosis of SI-NET recorded prior to the cohort start date (prevalent cases) were excluded.

As there is no precedent or consensus on an appropriate prediction horizon for a case-finding tool for SI-NET, and that SI-NET are rare, we sought to maximise the number of cases available for model development and evaluation. In preliminary analyses, using a time-to-event modelling framework with a prediction horizon of 2 years from cohort entry would lead to over 50% attrition of NET cases included for analysis (i.e. most individuals were diagnosed over 2 years after cohort entry). In order to maximise case numbers, and for the purposes of computational efficiency when developing multiple case-finding (diagnostic) models with hyperparameter tuning repeated within a cross-validation framework, the extracted cohort dataset was converted to a matched, weighted case-control dataset for model fitting. Cases were assigned an index date of the recorded date of NET diagnosis. Each case was matched with 100 non-cases from the same geographical region (*n* = 10). To account for time-varying contributions of follow-up from individuals during the cohort period, and possible trends in diagnostic modalities, non-cases were assigned an index date randomly drawn from a uniform distribution between their cohort entry date and their follow-up end date. For all individuals, predictor values were assigned at this index date—the most recently recorded values of BMI and smoking prior to/on the index date were used. To permit accurate estimation of model intercepts when fitting to case-control data (and therefore reliably predict probabilistic risks on unmatched data), participants were also assigned weights. Cases were assigned a weight of 1 and controls were assigned a weight equal to the inverse of the sampling fraction—these were used when fitting models (see below).

### Outcome and candidate predictor definitions

The outcome was defined as the presence of a recorded SNOMED/Read code for SI-NET (see **link to code** below). Predictors were defined by SNOMED clinical codes, with code lists developed and cross-checked by two clinicians with experience in EHR research (AKC & OB). Three categories of candidate predictors were based on clinical understanding and epidemiological evidence [14] and are summarised in Table 1. These were: factors associated generally with the risk of developing a gastrointestinal cancer (e.g. age, family history), symptoms or signs that could be attributable to an underlying SI-NET (e.g. abdominal pain), and features that reflect the diagnostic journey towards diagnosis or potential misdiagnoses (e.g. imaging or coeliac testing, and functional gastrointestinal disorder, respectively). Comorbidities were defined as being recorded in the primary care record at any point prior to the index date. For symptoms and investigations, these were defined as a recorded clinical code in the primary care record at any point in the 5 years prior to the index date—this was based on recent studies suggesting that NET patients may start consulting with their general practitioner up to 5 years prior to ultimate diagnosis [6, 7].

**Table 1:** Characteristics and predictor distributions in individuals with a recorded diagnosis of midgut NET and those that did not.

Parameter	NET cases *n* (column %)	Non-cases *n* (column %)
Total	382	11,719,560
Sex		
Male	198 (51.83%)	6,049,632 (51.62%)
Female	184 (48.17%)	5,668,705 (48.37%)
Other*	0 (0%)	1442 (0.01%)
Mean age at index date (SD)	62.71 (15.27)	45.22 (19.54)
Median age at index date (IQR)	64.84 (53.30–73.72)	41.84 (28.40–59.80)
Body mass index (kg/m^2^)		
Mean (SD)	27.13 (5.09)	26.16 (5.74)
Median (IQR)	26.8 (23.46–30.1)	25.3 (22.6–29.1)
Not recorded	133 (34.82%)	5,830,557 (49.75%)
Smoking status		
Never smoker	180 (47.12%)	5,690,551 (48.56%)
Ex-smoker	125 (32.72%)	2,158,669 (18.42%)
Light smoker (<10/d)	13 (3.40%)	321,213 (2.74%)
Moderate smoker (10–19/d)	<10	221,974 (1.89%)
Heavy smoker (20+/d)	<10	99,152 (0.85%)
Not recorded	53 (13.87%)	3,228,220 (27.55%)
Anaemia	32 (8.38%)	363,753 (3.10%)
Prior cholecystectomy	32 (8.38%)	217,700 (1.86%)
Recorded family history of gastrointestinal cancer	<10	54,398 (0.46%)
Functional gastrointestinal disorder	62 (16.23%)	573,481 (4.89%)
Inflammatory bowel disease	16 (4.19%)	108,674 (0.93%)
Peptic ulcer disease	13 (3.40%)	62,886 (0.54%)
Diabetes mellitus	41 (10.73%)	562,070 (4.80%)
Diverticular disease	51 (13.35%)	255,495 (2.18%)
Recent flushing	<10	59,401 (0.51%)
Recent abdominal pain	92 (24.08%)	767,719 (6.55%)
Recent bloating	<10	77,527 (0.66%)
Recent bowel change	20 (5.24%)	73,254 (0.63%)
Recent coeliac screen	<10	56,586 (0.48%)
Recent diarrhoea	80 (20.94%)	561,663 (4.79%)
Recent dyspepsia	35 (9.16%)	297,199 (2.54%)
Recent indigestion	27 (7.07%)	190,265 (1.62%)
Recent nausea/vomiting	12 (3.14%)	115,311 (0.98%)
Recent palpitations	14 (3.66%)	185,231 (1.58%)
Recent back pain	68 (17.80%)	1,202,407 (10.26%)
Recent weight loss	<10	80,287 (0.69%)
Recent endoscopy	69 (18.06%)	261,315 (2.23%)
Recent abdominal ultrasound	60 (15.71%)	595,750 (5.08%)
Recent abdominal CT/MRI	36 (9.42%)	211,813 (1.81%)

Status/measurements were the most recently recorded prior to the index date. Age corresponds to age at the diagnosis date (for cases) or index date (for non-cases).

Recent refers to a recorded clinical code within the 5 years prior to the index date.

*CT* computed tomography, *MRI* magnetic resonance imaging.

* = reflects the coding in the source database.

Fractional polynomials with up to two powers were used to model potential non-linearities between age and body mass index (BMI) and the outcome for the regression and penalised regression models. A closed-test procedure was used to identify polynomial terms that minimised the deviance [15]. Pre-specified interaction terms were between weight loss and BMI, age and diabetes, and age and functional gastrointestinal disorder.

### Missing data

There was incomplete recording of BMI and smoking status due to non-recording by the index date. This was handled using single imputation with chained equations due to computational considerations – the imputation model included all candidate predictors (including fractional polynomial terms), pre-specified interactions and the outcome, and imputation was performed separately for each region. This singly imputed dataset was used throughout all model development and evaluation steps.

### Model development and evaluation

All models were fit to the whole nested and weighted case-control dataset. Tenfold cross-validation was used to identify lambda values for the LASSO and ridge models that minimised the cross-validated deviance. These models were then refitted to the dataset with these lambda values.

Continuous variables were left unscaled for the XGBoost model, and categorical predictors were handled as dummy variables. Hyperparameter tuning with Bayesian Optimisation and 10-fold cross-validation was used to identify the configurations of the XGBoost parameters that maximised the cross-validated area under the curve (AUC). The final XGBoost model was then fit to the dataset with these hyperparameters.

The performance of each model was then assessed with internal-external cross-validation (IECV) using non-random dataset splitting by geographical region [12]. Our approach sought to support computational tractability by using the weighted, matched case-control data for model fitting (and repeated fitting during IECV), but using the entire unmatched dataset for model evaluation. By non-randomly splitting the whole dataset into geographically distinct units, this provides a stronger assessment of transportability to new settings than using a single random split, which would yield two sub-datasets with similar distributions of predictors [12, 16]. In the IECV process, one region was held out, the model refit to the matched, case-control data from all other regions—the case weights were applied at this stage. Then, the performance of that model was evaluated on full (unmatched) data for the held-out region. This was iterated so that all regions were used once as test sets. Region-level performance metrics (AUC, calibration slope, and calibration-in-the-large) were pooled with a random effects meta-analysis model (Hartug-Knapp-Sidik-Jonkmann approach [17]) to provide a pooled overall estimate, 95% confidence intervals and a 95% prediction interval. The latter provides an indication of the range of model performance if applied in a new, similar setting [16]. Cross-validation of lambda values of the penalised models and hyperparameter tuning for XGBoost was recapitulated in every iteration of IECV to provide a form of ‘nested’ cross-validation that avoided evaluating models on the same data used for tuning [18]. As the dataset was split by geographically distinct regions, there was no dependence across the ‘outer folds’, thus permitting meta-analytical pooling of region-level performance metrics.

Decision curve analysis [19] was used to explore the clinical utility (net benefit) associated with each model across a range of threshold probabilities. These analyses used the individual-level predictions generated for each participant obtained during IECV (i.e. when they were in the ‘held out’ region). The sensitivity and PPV of each model were assessed at different cut-offs of the predicted risk score distribution.

### Software and code

Data extraction used SQL. Analyses were conducted using Stata v17 and R, with analysis code available in the following repository: https://github.com/Mendelian/NETs_prediction_modelling.

### Study approval and conduct

Ethical approval for the OPCRD database for clinical research has been obtained from the NHS Health Research Authority (REC reference 20/EM/0148). This study was approved by the ADEPT committee (reference: PROTOCOL2318). The study is reported in accordance with TRIPOD guidance [20].

## Results

The final cohort comprised 11,719,942 individuals, of which 382 had a recorded diagnosis of SI-NET (0.003%). The cohort is summarised in terms of predictor distributions in Table 1.

Non-linearities were selected for age and BMI for the regression, LASSO and ridge regression models, with powers of [2, 3] and (0.5, 2), respectively. Supplementary Table 1 summarises the hyperparameter tuning space and final configurations of the XGBoost model.

### Model performance—summary performance metrics

Summary performance metrics for each model estimated after IECV are summarised in Table 2. The XGBoost model has the highest discrimination, with an AUC of 0.869 (95% CI: 0.841–0.898, 95% PI: 0.795–0.944], but confidence and prediction intervals around this metric overlapped between models. Calibration varied slightly with each approach, with the logistic and XGBoost models having slope values < 1 and > 1, respectively, but the magnitude of miscalibration on these summary measures was relatively slight.

**Table 2:** Comparison of performance metrics for each model, estimated using internal-external cross-validation.

Model	AUC (95% CI) [95% PI]	Calibration slope (95% CI) [95% PI]	Calibration-in-the-large (95% CI) [95% PI]
Logistic regression	0.833 (0.810–0.856) [0.783–0.883]	0.900 (0.845–0.955) [0.783–1.016]	−0.010 (−0.186–0.166) [−0.490–0.470]
LASSO regression	0.850 (0.827–0.873) [0.798–0.902]	0.976 (0.929–1.024) [0.880–1.073]	−0.010 (−0.186–0.166) [−0.495–0.167]
Ridge regression	0.842 (0.815–0.869) [0.772–0.912]	0.983 (0.934–1.031) [0.884–1.081]	−0.012 (−0.195–0.172) [−0.514–0.491]
XGBoost	0.869 (0.841–0.898) [0.795–0.944]	1.165 (1.088–1.243) [0.988–1.343]	0.010 (−0.164–0.185) [−0.462–0.483]

*AUC* area under the curve, 95% *CI* 95% confidence interval, 95% *PI* 95% prediction interval.

**Fig. 1:**
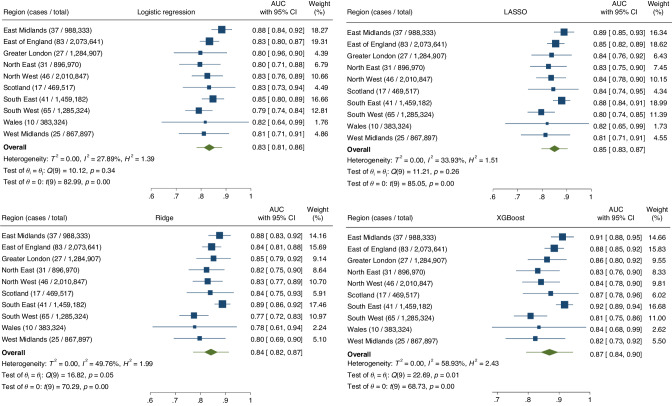
Forest plots summarising the region-level, pooled meta-estimates, confidence intervals and prediction intervals for the area under the curve for each model. Top left = logistic regression, top right = LASSO, bottom left = ridge regression, bottom right = XGBoost.

**Fig. 2:**
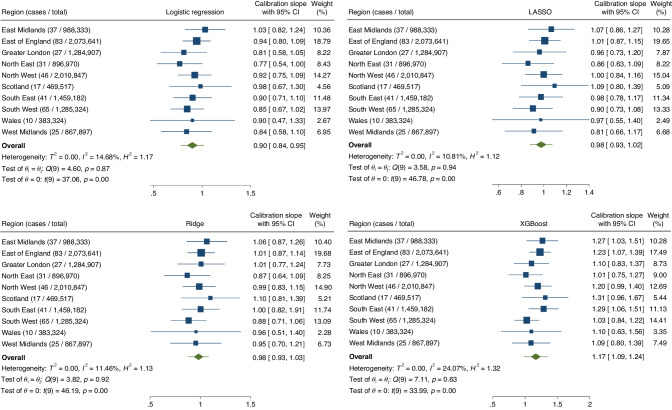
Forest plots summarising the region-level, pooled meta-estimates, confidence intervals and prediction intervals for the calibration slope for each model. Top left = logistic regression, top right = LASSO, bottom left = ridge regression, bottom right = XGBoost.

Region-level performance estimates for each model and their pooled meta-estimates are demonstrated in Figs. 1, 2, and Supplementary Fig. 1, for the AUC, calibration slope, and calibration-in-the-large, respectively. On analysing the performance metric results from IECV, we noted that the I^2^ for the XGBoost model was generally higher than for the regression/penalised regression-based approaches, and also visual inspection of the forest plots demonstrates more inter-regional variation. This could reflect lower model stability of more flexible algorithmic approaches to clinical prediction modelling in a rare disease setting.

### Model performance—sensitivity and PPV

Table 3 summarises the sensitivity of each model to capture SI-NET cases at different cut-offs of their predicted risk distributions. The general trend was that the XGBoost model had the highest percentage sensitivity at the thresholds examined. For example, the XGBoost model captured 18.84% of all NET cases in the highest 1% of predicted risks, and 45.55% of all NET cases within the highest 5%, suggesting potential for population-level risk stratification.

**Table 3:** Sensitivity of each model, defined as the percentage of all NET cases ‘captured’ within different cut-offs of their predicted risk distributions.

Risk score distribution cut-off	Logistic regression	LASSO regression	Ridge regression	XGBoost
1%	13.35%	14.13%	14.92%	18.84%
2%	21.20%	25.13%	21.47%	29.05%
3%	29.84%	31.94%	28.53%	37.43%
4%	35.60%	37.43%	34.03%	43.93%
5%	39.27%	41.10%	38.22%	45.55%
10%	52.09%	57.07%	52.09%	61.61%
20%	69.10%	70.68%	70.94%	75.92%
50%	92.15%	93.71%	91.99%	94.24%

In order to estimate the effect of model deployment, i.e. passive scanning of electronic health records and ‘flagging’ individuals at highest risk, we estimated the PPV across different definitions of ‘highest risk’ groups. As there needs to be cognisance of high clinician workload and pressure on referral systems in the context of a rare disease, we simulated this scenario by evaluating model PPV in the highest risk 0.001% to 0.005% groups; the latter represents 5 of every 10,000 patient records being flagged by an algorithm for clinical review. The XGBoost model typically outperformed other models, such as the PPV of 0.34% in the highest 1 in 10,000 individuals, but the results for PPV were modest (Supplementary Table 2**)**.

### Net benefit–decision analysis curves

**Fig. 3:**
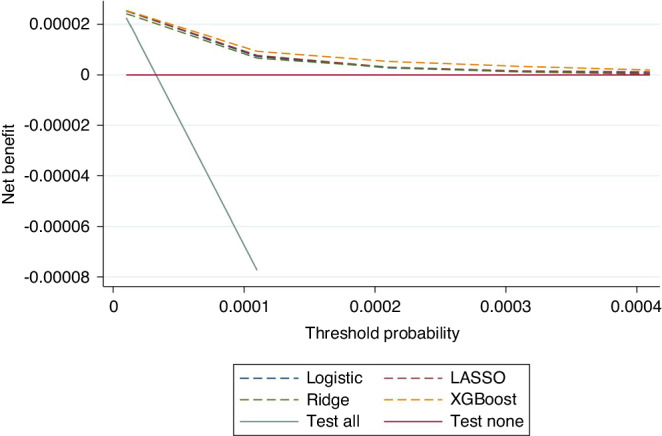
Net benefit analysis. Decision analysis curve comparing the clinical utility of all four models—these are also compared to the net benefit associated with ‘test all’ and ‘test none’ strategies.

The clinical utility of all 4 models was superior to the ‘test all’ and ‘test none’ strategies on decision curve analysis (Fig. 3).

## Conclusions

This study comparatively explored four different algorithmic approaches to developing a clinically useful model to predict the risk that an adult may have an underlying SI-NET, which could be used for case-finding to identify enriched sub-populations for referral to specialist/secondary care. The XGBoost model had the highest discrimination, the best sensitivity, and despite some mild miscalibration observed on summary metrics (slope, but not calibration-in-the-large), was associated with the highest clinical utility on decision curve analysis, albeit the net benefit curves did appear very similar across all 4 models.

Several studies have explored symptoms and diagnostic delays in NETs but these have mainly been based on patient-reported surveys [7, 21, 22]. Published hospital and primary care diagnostic pathway data in NETs is limited, with few studies assessing interventions to improve diagnosis [23]. Most clinical prediction modelling studies in NETs have focussed on predicting outcomes after diagnosis. However, one recent study used decision tree methodology on claims data to better understand clinical pathways to NET diagnosis, and also predict risks that a patient may have a NET [24]. However, that report has significant limitations in terms of its possible deployment in the target setting for the present study. First, there was no accounting for the over-representation of NET cases in the case-control sample, which means that the probabilistic estimates will be poorly calibrated. Second, that study only performed an apparent ‘validation’, and did not assess clinical utility or other key metrics [24]. Therefore, the present study is the first to develop a prediction tool to provide clinical decision support to target identification of SI-NETs in primary care.

Strengths of the present study include the size of the dataset used, which permitted ascertainment of sufficient case numbers to develop and evaluate the models, the use of an internal-external cross-validation framework to robustly assess and compare the performance of models developed using different techniques, and that the derivation data aligns with the target implementation of the models for their intended use case, representing a form of targeted validation [25]. The clinical coding, data availability and structured data fields within OPCRD represents data that are available at the point of care for general practitioners, and therefore the data that a model would theoretically utilise to make predictions would be available on deployment. Limitations of this study include the reliance of clinical practitioner coding for the predictors and outcome, and the inability to link the primary care to other secondary care datasets such as Hospital Episode Statistics or the national cancer registry (which will mean some under-ascertainment of cases), or additional linkages that integrated care services aim to compile (relevant to potential later model deployment). Further model evaluation in independent datasets that have such linkages should seek to quantify the yield in NET case ascertainment attainable. It is also worth noting that some ‘non-cases’ may have as-yet undiagnosed SI-NET, given the nature of the data. Misclassification bias may arise due to incorrect ascertainment of case status due to the reliance on clinical coding but also manifest as misclassification in terms of predictor value status (e.g. a patient actually underwent cholecystectomy but this was not coded in primary care notes). Due to the nature of the data used in this study - i.e. de-identified, coded primary care data, full manual record review using free text was not possible.

The study could have also considered EHR information in terms of not only a binary recorded/not recorded status for most predictors, but also the sequencing, frequency and timing of individual codes – this would be computationally intensive but could be of interest for future study either in this modelling scenario or for other case-finding tasks. Future projects may be able to model clinical free text with approaches such as unstructured data analysis, or leverage techniques from natural language processing, which could further boost model performance.

Whilst on summary metrics all models showed good discrimination and were associated with better net benefit than the logistically unfeasible ‘test all’ strategy on decision curve analysis, the performance of these tools as case-finding algorithms was far less optimistic when considering the PPV in the enriched sub-populations they could flag if deployed into EHR systems. This is a key consideration when developing prediction tools for rare conditions. Whilst this study sought to consider a broad range of potential predictors spanning risk factors, potential misdiagnoses, and other components of the ‘diagnostic odyssey’ in primary care reported by some individuals with NETs, ultimately the majority of features are non-specific. Features such as older age, multiple ‘gastrointestinal’ symptoms, and a positive family history of gastrointestinal cancer could lead to an individual being ‘flagged’ for a SI-NET, but may also be redolent of several other, far commoner conditions, such as a non-neuroendocrine neoplasm. Building on further research to explore the possibility of increasing specificity by more robustly modelling the temporality of EHR data, other work should also consider the overlap between the prediction model inputs and the risk of other, difficult-to-diagnose conditions. For example, a prediction tool with multinomial outputs for a collection of rarer, difficult-to-diagnose conditions with similar clinical features as NETs could serve to broaden the differential for a patient with unexplained symptoms that has had a colorectal carcinoma excluded, or be used to signpost such individuals to the most appropriate tests and services. Whichever approach is considered for clinical implementation would also need follow-on studies to estimate the clinical impacts and cost-effectiveness of algorithm-informed models of care – this should quantify the costs, utilities and pathway effects, and be contextualised with qualitative research regarding its acceptability to end users (e.g. general practitioners, secondary/tertiary care clinical teams, and patients). Such work is underway by this group.

In conclusion, by exploring four different prediction modelling approaches within an internal-external cross-validation framework, we identified that prediction models could have clinical usefulness in identifying undiagnosed people with SI-NETs in primary care. Whilst the XGBoost approach had the highest discrimination and highest net benefit, net benefit curves were rather similar, and the regression/penalised regression approaches could have benefits in terms of their transparency. As with other rare, long-term conditions, accurate case-finding approaches could open the possibility of promoting earlier diagnosis. In SI-NET, symptoms may be present for years prior to eventual diagnosis and cause multiple consultations in primary care. In order to robustly establish the proclivity of the case-finding tools reported in the present study to expedite diagnosis, ideally an external validation study would be performed that is: 1) aligned to the target population, and 2) explores model performance at different time points prior to NET diagnosis dates. This would provide useful information on the ‘lead time’ that could be offered by the model, and how risk model predictions evolve in the run-in to diagnosis. If attainable, being able to identify individuals with a NET earlier in the course of the disease and direct them towards appropriate investigation may improve the stage distribution at diagnosis and therefore improve clinical and patient outcomes. Given the low PPV, the optimal way to deploy this type of model in a healthcare system needs judicious consideration. For example, one could configure the target deployment population to be those within a specific age range (considering the higher age of NET cases vs non-NET cases), and/or excluding those with an existing diagnosis of cancer. It is possible that the best results could be obtained by integrating this model within a broader suite of clinical decision support tools to identify individuals engaging with primary care services that have undiagnosed conditions that include but are not limited to NET.

### Supplementary information

Supplementary tables and figure

## Data Availability

Due to data access licensing and data security, raw study data cannot be made available. Regulations regarding access to and use of the Optimum Patient Care Research Database are available here: https://www.opcrd.optimumpatientcare.org/licenses. Due to commercial considerations regarding intellectual property, the full regression models are not presented in this paper. These, and/or the final XGBoost model file could be made available to collaborators for the purposes of an external validation study after contacting the authorship team.
