# Supplementary material for: Identifying patients with undiagnosed small intestinal neuroendocrine tumours in primary care using statistical and machine learning: model development and validation study
Source: Br J Cancer. 2024 Jun 3;131(2):305–11. doi: 10.1038/s41416-024-02736-1 (PMC11263687; doi:10.1038/s41416-024-02736-1)
Supplement: Supplementary file 1 — Supplementary tables and figure [file 41416_2024_2736_MOESM1_ESM.docx]

| Hyperparameter | Search space | Final value selected during Bayesian optimisation |
| --- | --- | --- |
| Maximum tree depth | 1 to 10 | 6 |
| Learning rate (eta) | 0.0001 to 0.1 | 0.087 |
| Subsampling | 0.1 to 0.8 | 0.640 |
| Number of boosting rounds | 1 to 500 | 424 |
| Alpha regularisation | 0 to 20 | 3 |
| Gamma regularisation | 0 to 20 | 0 |
| Lambda regularisation | 0 to 20 | 9 |
| Column sampling (tree) | 0.1 to 0.8 | 0.499 |
| Column sampling (level) | 0.1 to 0.8 | 0.491 |

**Supplementary Table 1.** Hyperparameter search space and final configurations for the XGBoost model.

| Risk score distribution cut-off to generate a ‘flag’ | Logistic regression | LASSO regression | Ridge regression | XGBoost |
| --- | --- | --- | --- | --- |
| 0.001% | 0% | 0.26% | 0.09% | 0.34% |
| 0.002% | 0.04% | 0.17% | 0.13% | 0.26% |
| 0.003% | 0.06% | 0.14% | 0.11% | 0.28% |
| 0.004% | 0.09% | 0.15% | 0.09% | 0.26% |
| 0.005% | 0.12% | 0.12% | 0.07% | 0.20% |

**Supplementary Table 2.** Positive predictive value (PPV) of each model at selected cut-offs of the predicted risk distribution.


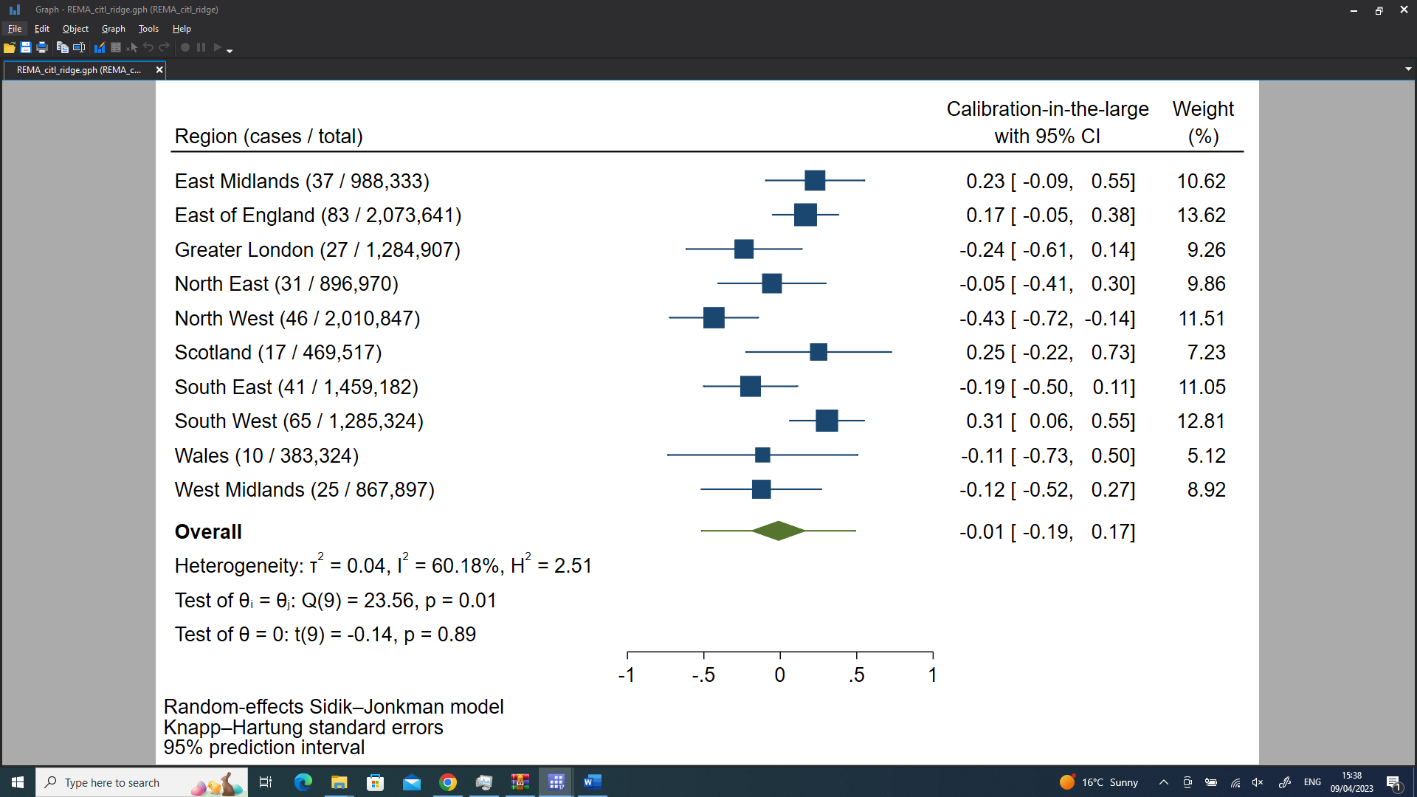

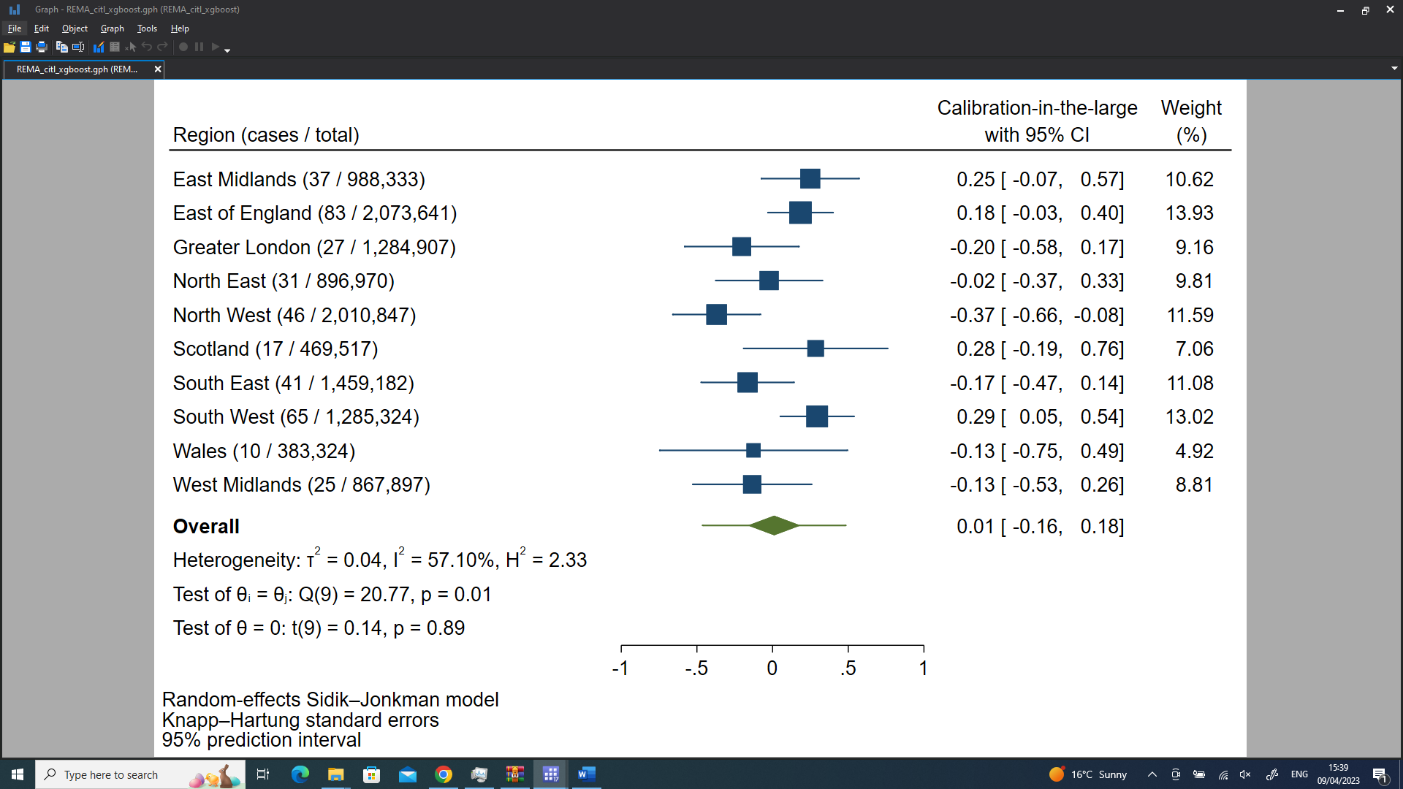

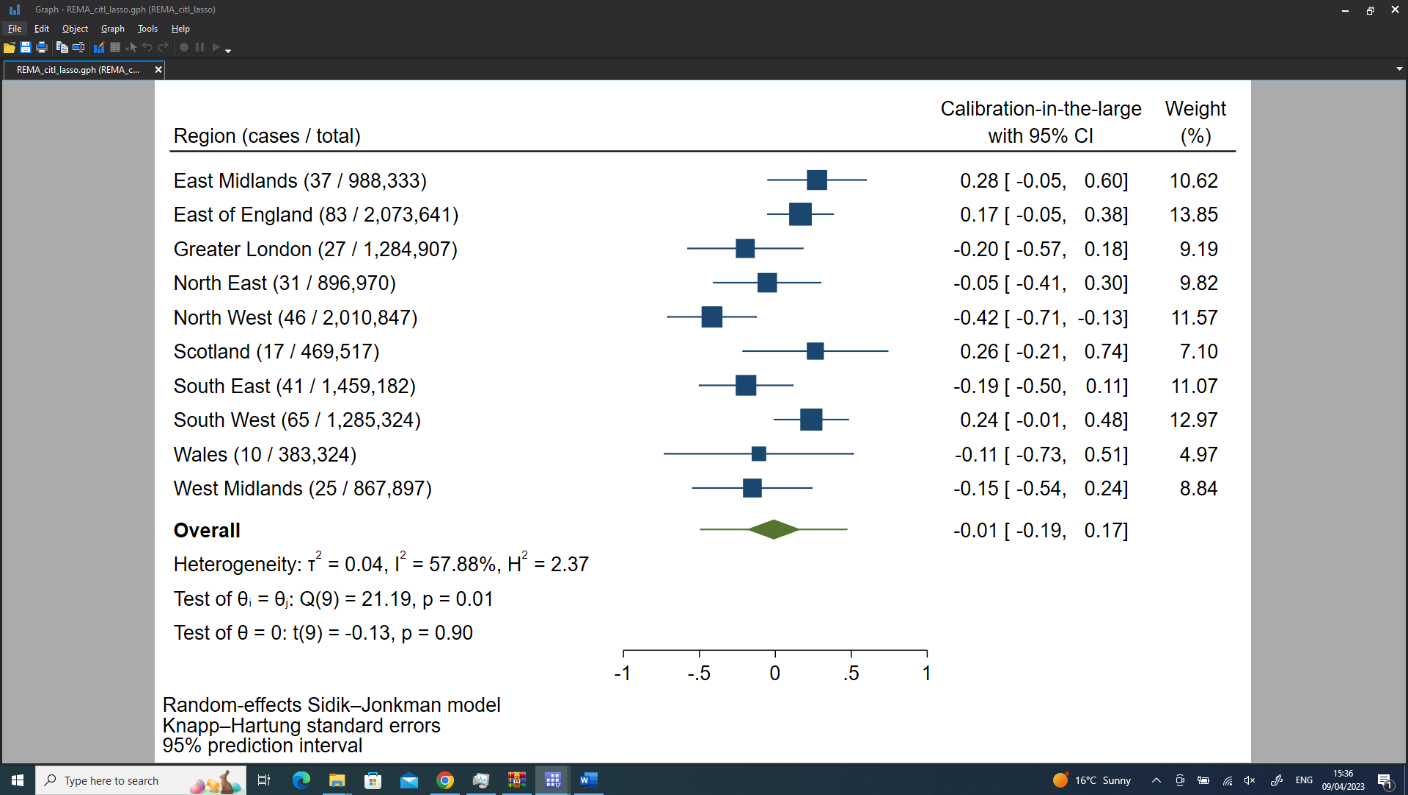

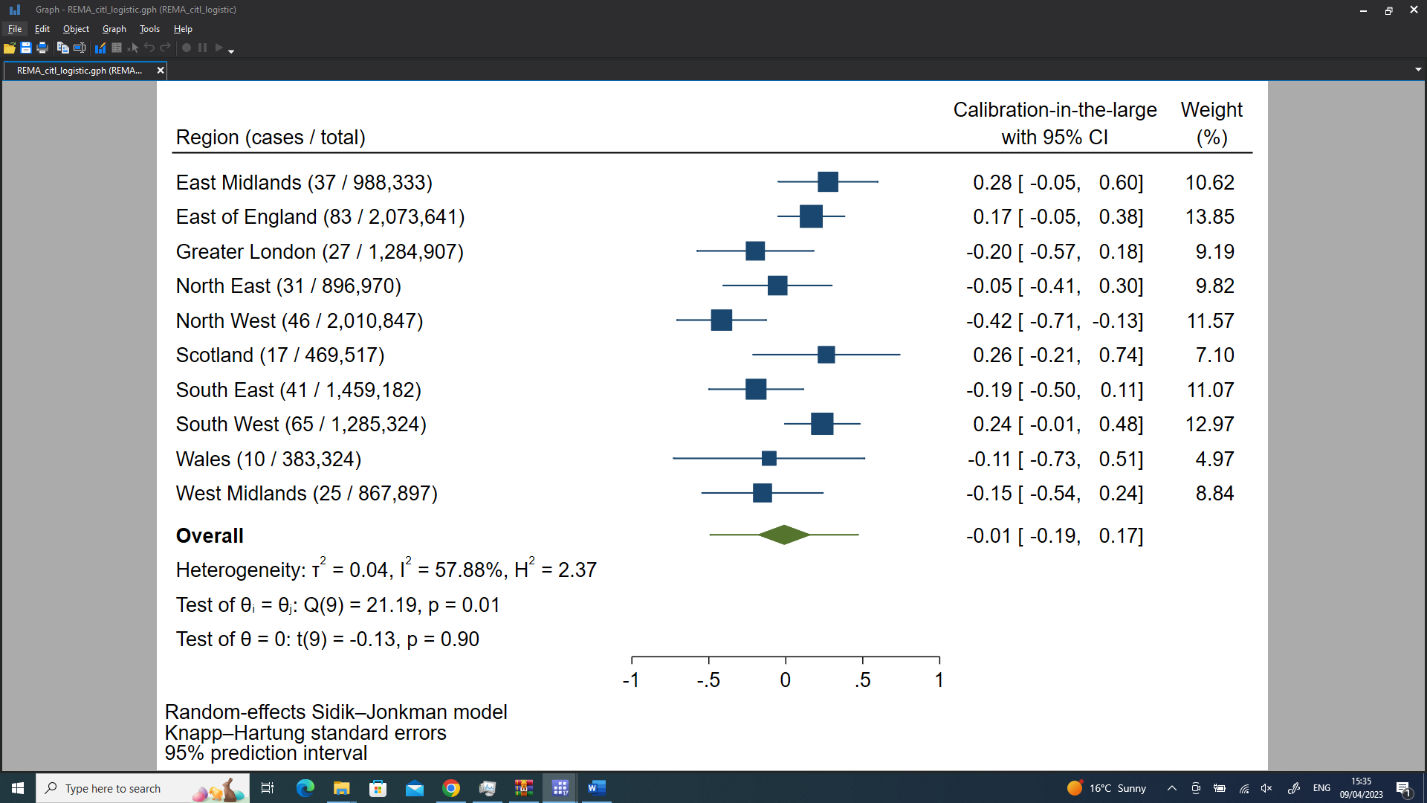


**Supplementary Figure 1.** Forest plots summarising the region-level, pooled meta-estimates, confidence intervals and prediction intervals for the calibration-in-the-large for each model. Top left = logistic regression, top right = LASSO, bottom left = ridge regression, bottom right = XGBoost.
